# Measuring the efficacy of a vaccine during an epidemic

**DOI:** 10.1371/journal.pone.0290652

**Published:** 2023-09-14

**Authors:** Antonio Scala, Pierpaolo Cavallo

**Affiliations:** 1 CNR-ISC, Applico Lab, Roma, Italy; 2 Centro Ricerche Enrico Fermi, Roma, Italy; 3 Big Data in Health Society, Roma, Italy; 4 Global Health Security Agenda - GHSA, Italy; 5 Department of Physics, University of Salerno, Fisciano, Italy; Universidade de Sao Paulo, BRAZIL

## Abstract

The urgency to develop vaccines during the COVID-19 pandemic has resulted in the acceleration of clinical trials. Specifically, a broad spectrum of efficacy levels has been reported for various vaccines based on phase III cohort studies. Our study demonstrates that conducting large cohort phase III clinical trials during the peak of an epidemic leads to a significant underestimation of vaccine efficacy, even in the absence of confounding factors. Furthermore, we find that this underestimation increases with the proportion of infectious individuals in the population during the experiment and the severity of the epidemic, as measured by its basic reproduction number.

## Introduction

The urgency driven by the COVID-19 pandemic has resulted in the implementation of clinical trials with procedures that adapt to the extraordinary circumstances [1] and the establishment of unprecedented public-private partnerships [2]. In particular, we have observed the case of vaccines that have reported widely different efficacies [3], varying from the ∼95% of Pfizer and Moderna (mRNA based), to the ∼70% of Astra-Zeneca or the ∼66% of Johnson & Johnson (viral vector-based). While mRNA-based vaccines, such as Pfizer-BioNTech and Moderna vaccines, instruct our cells directly to produce spike proteins, viral vector vaccines like AstraZeneca and Johnson & Johnson employ a harmless viral vector to deliver the genetic instructions for spike protein production to our cells. Once spike proteins are produced, our immune system recognizes them as foreign and mounts a response. This includes the production of antibodies that can bind to and neutralize the spike proteins, as well as the activation of T cells. These immune responses provide immunity against the virus. Given that all these vaccines induce an immune response by stimulating spike protein production, it is worth considering whether the variability in results could be influenced by differences in experimental conditions, such as the fraction of infectious individuals and the presence of variants. In this manuscript, we present findings that demonstrate how measuring vaccine efficacy at different stages of an epidemic’s progression can lead to a significant underestimation of the efficacy.

Vaccine efficacy is defined as one minus some measure of relative risk; according to the risk considered, several measures can be defined: efficacy for susceptibility to disease, for colonization, for progression, pathogenicity, infectiousness, indirect effects, population-level effects etc [4]. These measures require specialised and accurate datasets, sometimes with detailed information on the single contact experienced by the experimental cohorts. A vaccine of *efficacy* *ϵ* decreases the *transmission rate* by a factor *δ* = 1 − *ϵ*, i.e. a vaccinated person has a probability *δ* times lower of getting infected when coming into contact with an infectious individual; *δ* can be also be called the *relative risk* of vaccinated individuals [4]. We will employ a simpler characterization of the vaccine efficacy *ϵ* defined in terms of the transmission rate *β* of the epidemic. Thus, the transmission rate for vaccinated persons lowers from *β* to *β*^*V*^ = *δ* ⋅ *β* [5].

On the other hand, vaccine *effectiveness* *η* measures the real-world performance of a vaccine [6, 7], in contrast with efficacy that can be defined as the performance of an intervention under ideal and controlled circumstances. Factors concurring in a deviation of effectiveness from efficacy are multifaceted, and the implementation of effectiveness studies (especially challenging low- and middle-income countries) is affected by several confounding factors like age, socio-demographic factors (ethnicity/religion), geographical location, chronic disease and/or comorbidities and socio-economic status [8]. Thus, the same vaccine can have two different efficacies if tested or administered in two regions with different social contact patterns. In particular, a factor that has been mostly disregarded in large cohort (i.e. phase III) studies is the impact of the fraction of infectious individuals during the trial since in the past it had never occurred to develop and test a vaccine during an ongoing epidemic.

A key metric for the impact of a pandemic is the *basic reproduction* number $R_{0}$, measuring the expected number of people an individual can infect; $R_{0}$ can be calculated in terms of the transmission probability *β* and of the average lifetime *τ* of the infectious state as $R_{0}=\tau \cdot \beta$ [9]. The basic reproduction number allows us to estimate the *herd immunity threshold* (HIT), i.e. the fraction $\rho^{*}=1-1/R_{0}$ of immune individuals beyond which no epidemic overburst can happen [9]. The *efficacy* *ϵ* is paramount for estimating the *effective* fraction of people *ρ*^*V*^ = ρ^*^/*ϵ* to reach the HIT: the lower the efficacy, the higher the fraction of individuals to vaccinate.

The efficacy is not known a priori, but must be estimated through an experimental procedure. Overestimating *ϵ* would underestimate *ρ*^*V*^, with the danger of not reaching the HIT at the end of the vaccination campaign. Underestimating *ϵ* ensures that the fraction of vaccinated people is beyond the HIT; however, it increases both the costs and the duration of a vaccination campaign and—in extreme cases—it can lead to an estimate of the number of individuals to vaccinate beyond any practical possibility. As an example, if the fraction of kids in a population is *ρ*^*kids*^ but the vaccine cannot be administered to kids, *ρ*^*V*^ cannot be achieved if it is higher than 1 − *ρ*^*kids*^.

Reported efficacies are a measure of the reduction of the incidence of and outcome in a vaccinated group compared to an unvaccinated group *under optimal conditions in a clinical trial*. However, what happens if clinical trials are performed on large cohorts and during an epidemic so that it is possible that optimal conditions cannot be strictly enforced? As noted by Hallorane et al [10], to avoid that equivalent populations with the same transmission conditions could yield different efficacy estimates, the amount of exposure to infection should be taken into account either by study design or by mathematical modelling.

To isolate the effect of pursuing clinical trials during an ongoing epidemic, we will consider the theoretical case where no confounding factors [6, 7] intervene in the effectiveness—measured as the experimental ratio of infected individuals in a vaccinated and a placebo cohort—showing in long trials performed during an ongoing epidemic the *vaccine effectiveness* *η* underestimates the *vaccine efficacy* *ϵ*; such underestimation grows both with the fraction of infectious individuals *i* present in the population during the experiment and with the severity $R_{0}$ of the infection.

## Methods

### Vaccine efficacy

When the frequency of infective events in the susceptible individuals depends on the number of already affected individuals [11], the interpretation of the estimates of a vaccine efficacy can vary depending on the assumptions about the underlying dynamics [4]. Let’s assume that, to perform a double-blind evaluation of a vaccine’s efficacy, individuals have been divided into two cohorts *V* (the ones that have received the vaccines) and *P* (the ones that have received the placebo). Let’s also assume that the experimental protocol ensures that: (i) the individual in the cohorts are not in reciprocal contact (the ideal case is when infectious individuals in the cohorts remain reciprocally uncorrelated during the experiment); (ii) the infectious dynamics of the cohorts does not influence significantly the ongoing epidemics, i.e the size of the group is much smaller than the population and the observation time is much lower than the total time for the epidemic to evolve. Under these assumptions, infections come only from contacts with infectious individuals outside the cohorts. Thus, assuming full mixing, the probability of meeting an infectious individual is proportional to the fraction *i* of infectious individuals in the whole population, and the evolution of the fraction of susceptible individuals (i.e. not yet infected) in the *P*, *V* cohorts can be written as:

$$\begin{array}{c} \partial_{t}s_{P}=-\beta^{P}\cdot i\cdot s_{P}\;\;\;,\;\;\;\partial_{t}s_{V}=-\beta^{V}\cdot i\cdot s_{V} \end{array}$$ (1)

where the transmission probabilities are *β*^*P*^ = *β* for the placebo cohort and *β*^*V*^ = *δ* ⋅ *β* for the vaccine cohort, where *δ* = 1 − *ϵ*. Both equations can be solved yielding the solutions

$$\begin{array}{c} s_{P}=e^{-\beta^{P}c}\;\;\;,\;\;\;s_{V}=e^{-\beta^{V}c} \end{array}$$ (2)

where we indicate with $c=\bar{i}\;T=\int_{t}^{t+T}i\;d\tau$ the attack rate of the infection for the period [*t*, *t* + *T*], i.e the cumulative fraction of infectious individuals [9] during the trial. The corresponding attack rates for the cohorts will be *c*_*P*_ = 1 − *s*_*P*_ and *c*_*V*_ = 1 − *s*_*V*_; thus, we can rewrite Eq 9 as

$$\begin{array}{c} \eta =\frac{s_{V}-s_{P}}{1-s_{P}}=\frac{e^{-\delta \beta c}-e^{-\beta c}}{1-e^{-\beta c}} \end{array}$$ (3)

that tells us that the observed effectiveness *η* will depend on the attack rate relative to the observation period; notice that such expression is in accordance with the results of [12]. For small values of *βc*, it is possible to expand Eq 3:

$$\begin{array}{c} \eta =\epsilon [1-\frac{\delta }{2}\beta c+O\left(\beta^{2}c^{2}\right)] \end{array}$$ (4)

i.e. there is already a negative correction to the estimate of *ϵ* by *η* that is proportional to *δ* even for small values of *c* (i.e. when the average number of infectious $\bar{i}$ is small). Also the expansion to the second order

$$\begin{array}{c} \eta =\epsilon [1-\frac{\delta }{2}(\beta c+\frac{1-2\epsilon }{6}\beta^{2}c^{2})+O\left(\beta^{3}c^{3}\right)] \end{array}$$ (5)

retains the same behaviour, since the quadratic term is still negative up to very low efficacy *ϵ* = 0.5 and the corrections decrease proportionally to *δ*: the higher the efficacy, the better the effectiveness *η* estimates the efficacy *ϵ*.

### SIR model

To estimate *c*, it would be necessary to have accurate data on the fraction of infectious individuals during an epidemic, like the one obtained by testing campaigns. In cases like COVID19, where data are scarce and the understanding of the epidemic is still an ongoing process, it is useful to rely on mathematical models whose parameters are tuned to the epidemic’s dynamics. For its simplicity and for the few parameters needed, we will use the basic *SIR* model. While the *SIR* model is not the best model for accurate scenario forecasting, it captures the overall behaviour of an epidemic with a minimal amount of parameters; thus, it is often used in the first stages of an epidemic when data are still scarce. On the same footing, we will use the *SIR* model to have an *order-of-magnitude* estimate of the effects on the measurement of the effectiveness during an ongoing epidemic.

In the *SIR* model, the population is divided into three groups *S*,*I*,*R* corresponding to different stages of an infection: *S* corresponds to susceptible individuals, *I* to infectious and *R* to recovered individuals. Indicating with lowercase letters (i.e. *s*, *i*, *r*) the fractions of individuals in a given class, the epidemic is described by the equations

$$\begin{array}{c} \partial_{t}s=-\beta si\;\;\;,\;\;\;\partial_{t}i=\beta si-i/\tau \;\;\;,\;\;\;\partial_{t}r=i/\tau \end{array}$$ (6)

where *β* is the infection rate and *τ* is the average duration of the infectious period.

For the *SIR* model, since $\partial \;\text{ln}\;s=-R_{0}r$ [13], it is possible to derive the closed solution $\;\text{ln}\;s=-R_{0}\;r$ for a free epidemic starting from *s*(*t* = 0) = 1, *r*(*t* = 0) = 0; thus, since *i* is maximum when $s=1/R_{0}$ and at this value $r=\;\text{ln}\;R_{0}/R_{0}$, we can explicitly calculate the value of *i*^*max*^ from *i* + *s* + *r* = 1

$$\begin{array}{c} i^{max}=1-\frac{1+\;\text{ln}\;R_{0}}{R_{0}} \end{array}$$ (7)

showing that in *SIR* models the maximum fraction of infectious grows as expected with the basic reproduction number following a simple relation with $R_{0}$.

### Stochastic estimates of the efficiency

While deterministic equations for epidemic dynamics can be a good approximation when the population is large and as soon as there is an extensive number (even if the fraction is small) of infectious [9], in the case of medical experiments cohorts are seldom large enough to disregard statistical fluctuations in the observations. Apart from particular cases like systems with critical points [14], relative fluctuations for a system of *n* individuals are of the order $1/\sqrt{n}$. Thus, while for equations like *SIR*’s—describing populations of a size of the order of the inhabitants of a nation—we can disregard fluctuations and we can thus consider *i* as a good proxy for the evolution of the fraction of infectious, Eq 1 does not allow to check for the importance of fluctuations in the experimental setting when the number of cohorts’ patients *n* is not so large. As an example, cohorts of size *n* ∼ 10000 are expected to yield relative errors of order ∼1%.

**Fig 1:**
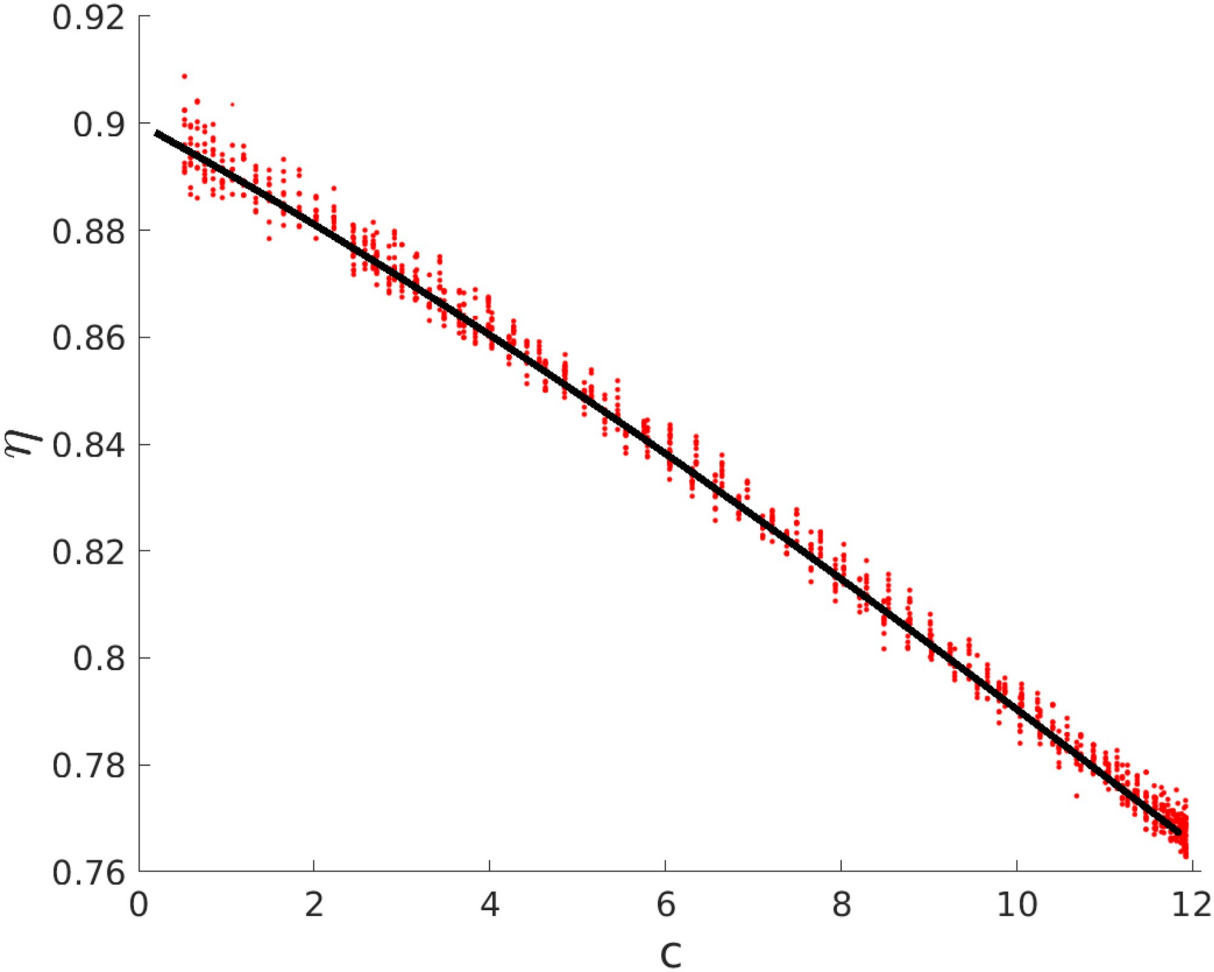
Measured effectiveness *η* (Eq 9) versus the attack rate *c*. The epidemic is modelled with a *SIR* model with basic reproduction number $R_{0}=3$ and mean infectious period *τ* = 15 days corresponding to a transmission rate *β* = 0.2 days^−1^. The continuous black line corresponds to the expected values of *η* (Eq 3) for trials of a duration *T* = 2 months and real efficacy *ϵ* = 0.90. Curves are obtained by varying the initial time *t* of the trial; thus, each *c* corresponds to a period [*t*, *t* + *T*]. Lower values of *c* correspond to the initial and final phases of the epidemics where the fraction of infectious individuals is low, while high values of *c* correspond to experiments performed near the peak of the epidemic. We observe that *η* is affected by a systematic error (i.e. *η* < *ϵ*) that makes it underestimate the real efficacy *ϵ*; when the fraction of infectious individuals is high, the error is larger, while when it is low, *η* ≈ *ϵ* and the error is proportional to *βc* (see Eq 4). To evaluate the statistical errors, we model the process of getting infected by a stochastic process (Eq 8) and simulate possible values of *η* for cohorts of *n* = 4 × 10^4^ individuals, i.e. of a size of the same order of the Pfizer trial [16]). As expected, the results of the stochastic simulations (red dots in the figure) fall in a region with a distance of order 10^−2^ around the theoretical curve of Eq 3, i.e. a region of order $\times 1/\sqrt{n}$ as expected for a trial with cohorts of independent, non-interacting individuals.

To estimate such statistical fluctuations, we employ a simplified stochastic approach. Since the *V*, *P* experimental cohorts consist of independent and uncorrelated individuals, the infection rates *β*^*X*^*i*, *X* ∈ {*V*, *P*} of Eq 1 can be interpreted as independent Poisson rates where each individual in the cohorts has a probability −*β*^*X*^*i* per unit time to become infected. In a time interval Δ*t* small enough that *i* can be considered constant, the number of infections suffered by a population of *S*^*X*^ individuals will thus follow a Poisson distribution of mean *β*^*X*^*iS*^*X*^Δ*t*; thus, the infectious dynamics for the experimental cohorts can be simulated as

$$\begin{array}{c} S^{X}\left(t+\Delta t\right)=S^{X}\left(t\right)-PoissRand(\beta^{X}\cdot i\left(t\right)\cdot S^{X}\left(t\right)\cdot \Delta t) \end{array}$$ (8)

where *X* ∈ {*V*, *P*} and **PoissRand**(*x*) generates a random integer number Poisson distributed with rate parameter *x*. Such an approach has been applied to estimate the fluctuations reported in Fig 1.

## Results

The effectiveness *η* of the vaccine is measured by confronting the infections occurring into two observed groups (also called *cohorts* in the medical language), one that has been vaccinated (cohort *V* of size *N*_*V*_) and one that got a placebo (cohort *P* of size *N*_*P*_) [5]. The distribution of the traits (age, census, medical history, etc) of both cohorts must be representative of the whole population; moreover, (i) the individuals of both cohorts should be distributed in the population so as not to have contacts among themselves (to avoid spurious correlations), (ii) the observation period *T* should be long enough to have a statistically significant number of observed cases of infections. Thus, the effectiveness *η* is estimated as the proportion of persons in the placebo group of a vaccine trial who would not have become ill if they had received the vaccine [5]. Let us indicate with *A*^*V*^ and *A*^*P*^ the number of cases in the vaccinated and placebo cohorts, respectively, at the end of the study. Suppose that we are analysing a large trial (like a phase III study) where, given the number of participants, it is not possible to have detailed information about their contacts. If we indicate with *c*_*P*_ = *A*_*P*_/*N*_*P*_ and *c*_*V*_ = *A*_*V*_/*N*_*V*_ the attack rates (or cumulative incidence), i.e. the fraction of individuals that get infectious during the trial, the vaccine efficacy can be expressed as [4]

$$\begin{array}{c} \eta =1-\frac{c_{V}}{c_{P}} \end{array}$$ (9)

however, we must bear in mind that interpreting efficacy estimates is a multifaceted issue [4].

Notice that Eq 9 could be a good estimate of the vaccine efficacy *ϵ* if the observed cohorts are under controlled clinical trial conditions [4]; in the case of large phase III studies, this is not the case: in particular, if phase III studies are performed *during* an epidemic, the measure of *η* could depend on the fraction of infectious individuals in the population.

The effectiveness *η* aims to be a proxy (in statistical language—an estimator) of the *real efficacy* *ϵ* of the vaccine. In the following, due to the nature of a phase III experiment (large numbers of individuals not subject to clinical trial conditions), we will assume that cohort individuals are in contact with the infected population. Since the number of individuals in the cohorts is much smaller than the population, we will also assume that the individuals in the *V*, *P* cohorts are uncorrelated as required from the experimental protocol. Finally, we will assume that the dynamics of the cohorts do not influence significantly the ongoing epidemics: this is true if the size of the group is much smaller than the population and if the observation time is much lower than the total duration of the epidemic. Under such assumption, it is possible to derive an explicit formula (Eq 3) for the final values of the effectiveness *η* in terms of the relative attack rate *c*, of the transmission rate *β* and of the relative risk *δ*. Since the attack rate can be expressed as $c=\bar{i}\;T$, we can see that the key drivers are the length *T* of the experiment and the average fraction $\bar{i}$ of infectious during the period *T*.

In the following, we will show the results for SIR models with parameters in the range of COVID19 estimates; in particular, we will assume that the infectious period is *τ* = 15 days and $R_{0}$ is in the range [2.5, 6.0] [15]; however, since Eq 3 does not depend on the details of the dynamics, the results are expected to be robust in respect of the epidemic model employed.

In Fig 1, we show how the expected estimates of *η* (Eq 3) for a real efficacy *ϵ* = 0.90 decrease as a function of the attack rate *c*. The basic reproduction number is fixed to be $R_{0}=3$, while the duration of the trial is fixed to be *T* = 4*τ*, i.e. a period of ≈2 months. Lower values of *c* correspond to the initial and final phases of the epidemics where $\bar{i}\ll 1$, while high values of *c* correspond to experiments performed near the peak of the epidemic. We observe that *η* tends to underestimate *ϵ* more when the fraction of infectious individuals is high, while *η* ≈ *ϵ* in the initial phases where $\bar{i}\ll 1$; in particular, in this regimes, the corrections to *η* (i.e. the systematic errors introduced by using Eq 9) are small and proportional to the attack rate during the trial (see Eq 4).

When the number of individuals is small, the process of getting infected is better described by a stochastic process. We thus perform stochastic simulations of the experiments (see Methods) for cohorts of *n* = 4 × 10^4^ individuals, i.e. of a size of the same order of the Pfizer trial [16]). In Fig 1 we show as red dots the results of stochastic simulations of the trial; the results of the stochastic simulation fall in a region with a relative distance of order 10^−2^ around the theoretical curve of Eq 3, i.e in a region of order $\pm 1/\sqrt{n}$ as expected for a trial with cohorts of independent, non-interacting individuals.

**Fig 2:**
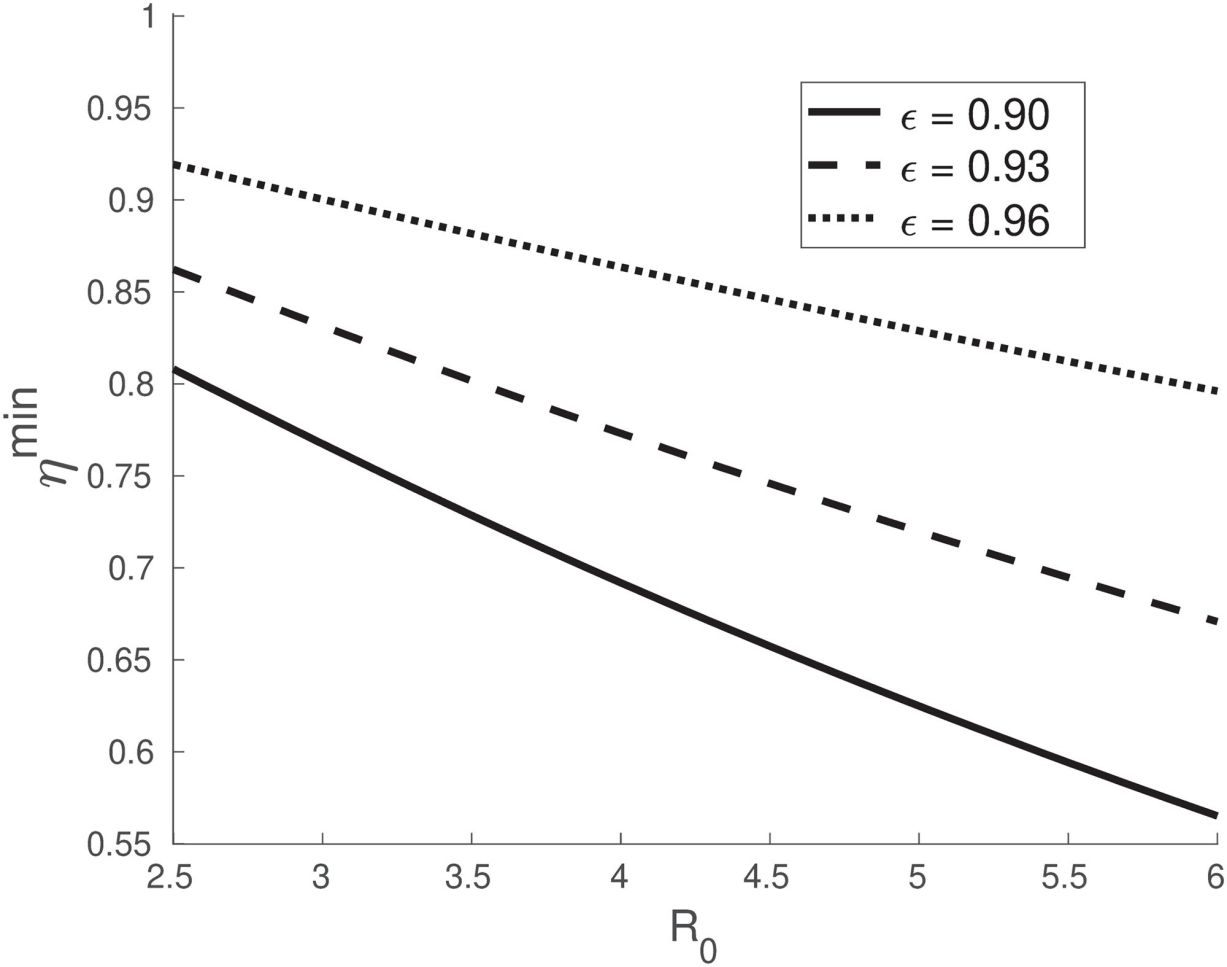
Minimum efficacy vs basic reproduction number. According to Eq 3, the measured effectiveness *η* (Eq 9) reaches a minimum *η*^*min*^ for clinical trials near the epidemic peak. The figure reports the theoretical values of the worst effectiveness’ estimate *η*^*min*^ versus the basic reproduction number $R_{0}$ when modelling the epidemic with a *SIR* of mean infectious period *τ* = 15 days and considering clinical trials of length *T* = 2 months. The three curves correspond to a true efficacy of *ϵ* = 0.90 (continuous line), *ϵ* = 0.93 (dashed line) and *ϵ* = 0.96 (dotted line). The curves show that the lower a vaccine’s efficacy *ϵ*, the worse it is underestimated by the effectiveness *η* (Eq 9).

Since the maximum number of infectious individuals is an increasing function of $R_{0}$ (see Eq 7), the maximum attainable value of *c* also increases with $R_{0}$; thus, the worst estimated effectiveness *η*^*min*^ (i.e. at the infectious peak) must also be a decreasing function of $R_{0}$. In Fig 2, we show that this is the case by plotting *η*^*min*^ as a function of $R_{0}$ for *ϵ* = 0.90, 0.93, 0.96 and for a duration of the experiment of 4*τ*, i.e. *T* ≈ 2 months. Notice that, if the vaccine has a higher efficacy *ϵ*, then *η* better estimates it: as an example, for $R_{0}=3$ (a value that has been estimated for COVID19 in France [17]), to an efficacy *ϵ* = 0.96 corresponds an effectiveness as low as *η*^*min*^ ≈ 0.90 (i.e. Eq 9 introduces a systematic error up to ∼6%), while to an efficacy *ϵ* = 0.90 corresponds an effectiveness as low as *η*^*min*^ ≈ 0.77 (i.e. Eq 9 introduces a systematic error up to ∼15%).

**Fig 3:**
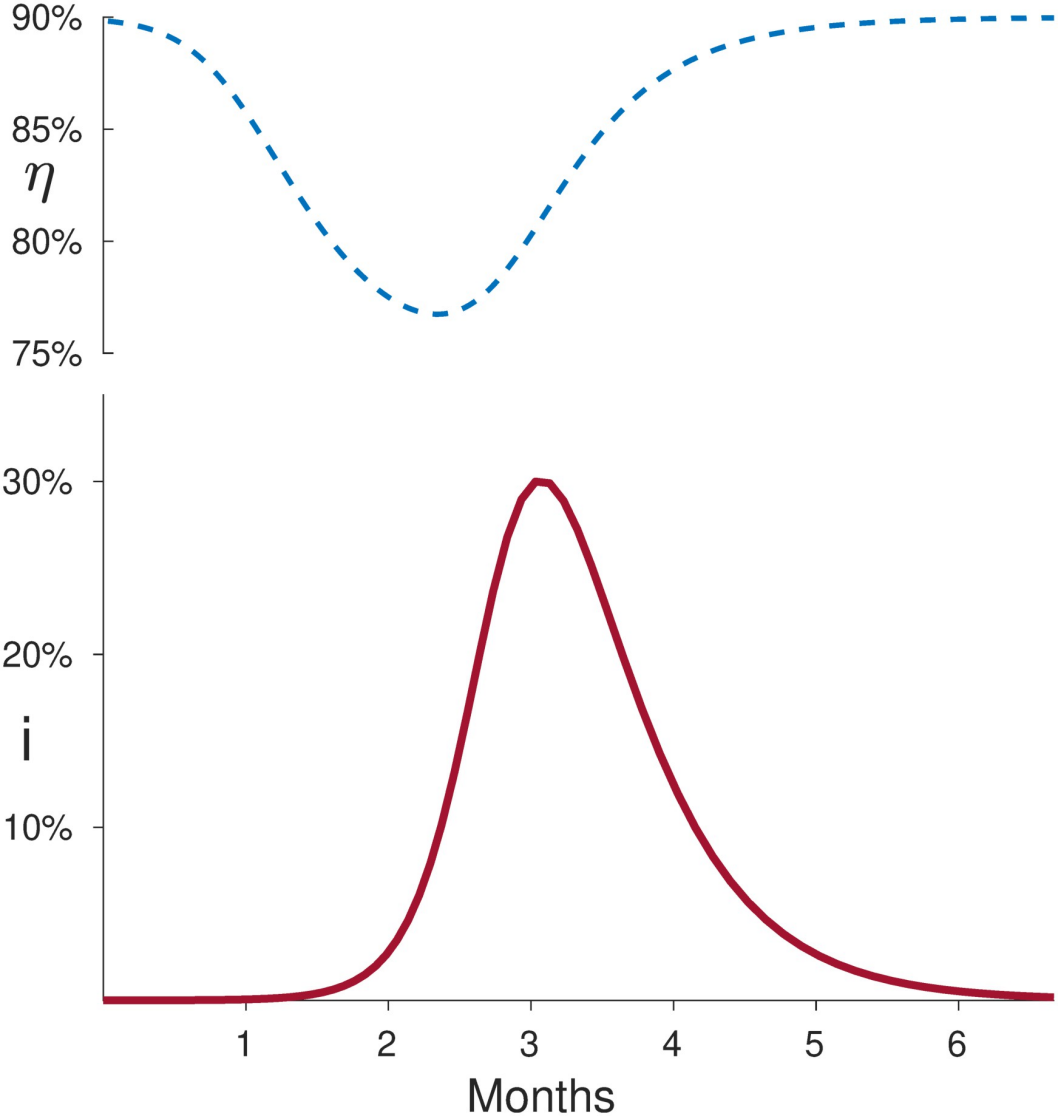
Measured effectiveness and fraction of infected individuals versus time. We consider an *SIR* model with $R_{0}=3$ and *τ* = 15 days. Upper panel: the effectiveness *η*(*t*) measured as Eq 9 on a series of *T* = 2 months trials starting at different times *t* for a vaccine with *true* efficacy *ϵ* = 90%. As expected, *η*(*t*)∼*ϵ* when the initial fraction *i* of infected is very low. *η*(*t*) decreases as *i* grows and reaches a minimum during the infection peak. In fact, for the same number of infected at the beginning of a trial, the measured effectiveness will be lower if the epidemic is growing since the attack rate in Eq 9 will be higher.

Finally, in Fig 3, we show an example of the time-dependence of the measured effectiveness *η* in the case of a *SIR* model with $R_{0}=3$ and *τ* = 15 days. We then consider *η*(*t*) as the effectiveness measured as Eq 9 on a series of *T* = 2 months trials starting at different times *t* for a vaccine with true efficacy *ϵ* = 90%. As expected, *η*(*t*)∼*ϵ* when the initial fraction *i* of infected is very low, *η*(*t*) decreases when *i* grows and reaches before during the infection peak. In fact, for the same number of infected at the beginning of a trial, the measured effectiveness will be lower if the epidemic is growing since the attack rate in Eq 9 will be higher.

## Discussion

The rush to develop COVID-19 vaccines has compelled governments and developers to establish new standards for robust human clinical trials. It has become evident that methodological challenges in these trials can result in unrepresentative data, and communication errors can further contribute to vaccine hesitancy [18].

Assessing vaccine efficacy necessitates a comprehensive understanding of the trial participants’ health status and their contacts. However, this level of information is rarely available, except in early-stage phase I and II studies, where smaller groups in controlled settings allow for more in-depth insights into the vaccine’s effects. These studies can help separate the indirect effects caused by a growing number of infections in the overall population [19] and distinguish the vaccine’s efficacy in protecting individuals from the disease versus its effectiveness in reducing transmission from vaccinated individuals [4]. Nevertheless, even with a detailed understanding of trial dynamics, phase I and II statistics often lack the necessary precision. On the other hand, phase III trials involve a large number of participants, enabling more accurate estimations at the cost of insufficient detailed information to discern different types of efficacy and evaluate indirect effects. In this regard, leveraging digital contact tracing [20] along with national health service medical records can be crucial throughout various stages of a pandemic crisis. These measures aid in early detection, outbreak isolation [21], calibration of pharmaceutical and non-pharmaceutical interventions [13, 22], phase III efficacy estimation for rapidly developed vaccines, and phase IV evaluation of side effects once distributed among the population [18]. Nonetheless, we have demonstrated that collecting more reliable but less granular data, such as the fraction of infectious individuals in a population, would significantly improve the interpretation of medical trial results.

Indeed, we have illustrated how even a rudimentary understanding of epidemic data, such as estimates of the fraction of infectious individuals over time, can help rectify efficacy estimates from measured effectiveness during phase III (and potentially phase IV). Of course, more detailed data can provide insights into the impact of contact heterogeneity [22–24] on epidemic dynamics, vaccine efficacy, and herd immunity thresholds. Digital tracing data, in particular, would be invaluable for considering variations in contact patterns influenced by human behaviour. For instance, vaccinated individuals may modify their habits if they believe the vaccine offers protection. Therefore, without detailed contact information, these behavioural changes could introduce systematic biases in efficacy trials [10]. Overall, understanding and quantifying the effects of human behaviour are pivotal for effective policies regarding non-pharmaceutical interventions and vaccination strategies [25, 26].

## Conclusions

Many factors impact the efficacy of a vaccine: from population-specific genetic characteristics to partial immunity acquired from previous infections, or even the development of variants during the epidemic: something that, given the duration of the still ongoing pandemic period, has occurred for COVID19. However, our study concentrates on the systematic underestimation of vaccine efficacy *ϵ* by the estimated vaccine effectiveness *η* in large cohort studies due to the presence of a high number of infectious individuals in the population. Since vaccines have never been produced, tested and experimented in such exceptional circumstances as the ones that recently occurred during COVID19, such an issue has not been fully addressed before.

For the sake of simplicity, we have employed a classical epidemiological model with realistic parameters to understand the order of magnitude of the systematic error in efficacy estimates; however, most models of epidemics do not differentiate between infection and disease, while there are cases where that the relation between the biological efficacy of the vaccine and its efficacy as measured by clinical trials is complex and multi-factorial [4, 27, 28]. Since disease (i.e. observable traits) is what drives behaviour, this is an issue that should be pursued further when modelling epidemics.

In the case of COVID19, age is a key factor with respect to the incidence of severe cases and/or mortality; in fact, efficacy estimates in phase III studies consider the effects of age [16, 29]. In such a case, we will have that effectiveness will be a larger underestimate of efficacy in the subgroups where the latter is lower. Moreover, since we have experienced that the COVID19 vaccine efficacy is time dependent and depends on the number of the doses [30], longitudinal studies should be planned in advance to detail the history-dependence of vaccine efficacy.

Finally, we notice that in the vaccine trials that have occurred, it has been observed that antibodies in the vaccinated individuals take time to develop [16, 29]; thus, if also the efficacy of a vaccine in a single individual grows with time, an extra bias could be introduced in efficacy and effectiveness measurements. In particular, if the trial occurs when the number of infectious is growing, the protection is low at the beginning of the trial, when the probability of getting infected is lower; on the contrary, if the epidemic is decreasing, the vaccine protects less at the beginning, i.e. just when the probability of getting infected is higher. Thus, for two trials—one before the epidemic peak, the other after—with identical time spans and attack rates, we expect a lower estimate of the vaccine efficacy (i.e. a larger systematic error) for the trial in the decreasing phase.

## Supporting information

S1 Graphical abstract (TIF)
