# Supplementary figures and images for: Measuring the efficacy of a vaccine during an epidemic
Source: PLoS One. 2023 Sep 14;18(9):e0290652. doi: 10.1371/journal.pone.0290652 (PMC10501570; doi:10.1371/journal.pone.0290652)

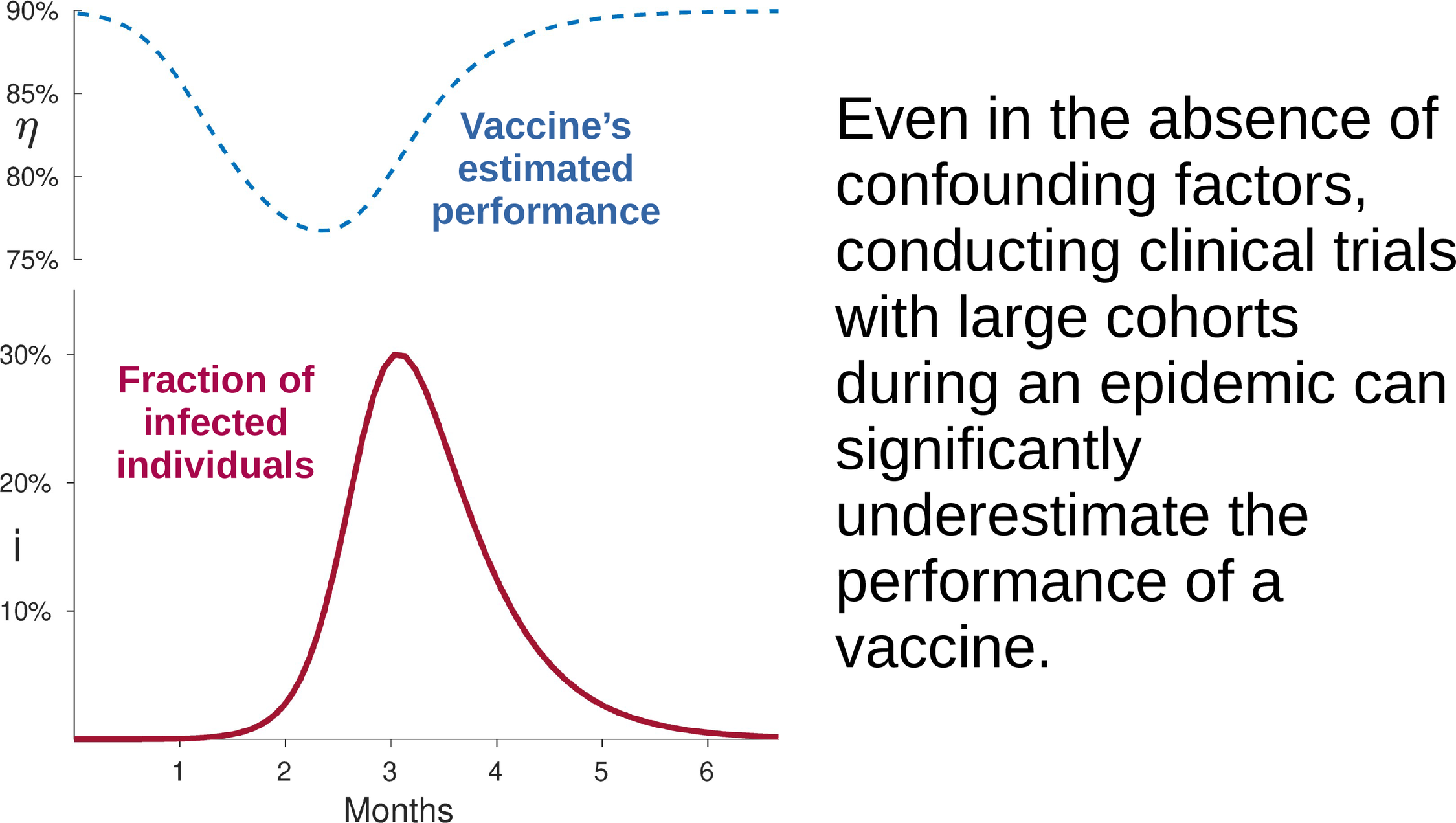

Supplement: S1 Graphical abstract — (TIF) [file pone.0290652.s001.tif]
